# Critical synchronization and 1/*f* noise in inhibitory/excitatory rich-club neural networks

**DOI:** 10.1038/s41598-018-37920-w

**Published:** 2019-02-04

**Authors:** Daniel Aguilar-Velázquez, Lev Guzmán-Vargas

**Affiliations:** Unidad Profesional Interdisciplinaria en Ingeniería y Tecnologías Avanzadas, Instituto Politécnico Nacional, Av. IPN No. 2580, L. Ticomán, Ciudad de México 07340 Mexico

## Abstract

In recent years, diverse studies have reported that different brain regions, which are internally densely connected, are also highly connected to each other. This configuration seems to play a key role in integrating and interchanging information between brain areas. Also, changes in the rich-club connectivity and the shift from inhibitory to excitatory behavior of hub neurons have been associated with several diseases. However, there is not a clear understanding about the role of the proportion of inhibitory/excitatory hub neurons, the dynamic consequences of rich-club disconnection, and hub inhibitory/excitatory shifts. Here, we study the synchronization and temporal correlations in the neural Izhikevich model, which comprises excitatory and inhibitory neurons located in a scale-free hierarchical network with rich-club connectivity. We evaluated the temporal autocorrelations and global synchronization dynamics displayed by the system in terms of rich-club connectivity and hub inhibitory/excitatory population. We evaluated the synchrony between pairs of sets of neurons by means of the global lability synchronization, based on the rate of change in the total number of synchronized signals. The results show that for a wide range of excitatory/inhibitory hub ratios the network displays 1/*f* dynamics with critical synchronization that is concordant with numerous health brain registers, while a network configuration with a vast majority of excitatory hubs mostly exhibits short-term autocorrelations with numerous large avalanches. Furthermore, rich-club connectivity promotes the increase of the global lability of synchrony and the temporal persistence of the system.

## Introduction

In recent years, the structure (human connectome) and dynamics of human brain networks have started to be unveiled by means of numerous neuroimaging techniques. The structural properties of the human connectome are frequently described by a short average path-length between nodes^1^, high clustering^2,3^, hierarchical modularity^4^, and a heavy-tailed distribution of connectivity, with highly connected brain regions or brain hubs^5,6^. Brain hubs are strongly interconnected among them, forming a rich-club^7^. Diverse studies have reported that brain hubs play a key role in integrating and interchanging information between diverse brain regions^8^. Two types of brain hubs are recognized: provincials and connectors. Provincial hubs show connections within a single cluster, while connector hubs are mostly connected with two or more clusters^7^. Connector hubs are involved in complex cognitive tasks, while provincial hubs are involved in specific tasks^8^. Many brain diseases are associated with a disruption in structural and functional connectivity: either alterations in the path-length average and clustering (small-world properties)^9–12^, or the affection of brain hub regions^13–17^. In addition, disturbed rich-club connectivity promotes several neural pathologies^18–23^. For instance, Bonifazi *et al*.^24^ found that the activity of hub neurons can perturb the entire network’s dynamics and the existence of highly connected neurons play a key role in the synchronization; these hubs neurons are commonly assumed to be inhibitory neurons (GABAergic interneurons). Inhibitory neurons comprise the 15–20% of the population, while the majority 80–85% are excitatory neurons (Glutamatergic cells)^25,26^. Inhibitory neurons offer stability to neural networks^27^, and facilitate the corrected routed of excitatory paths^28^. Moreover, they are involved in brain development, exciting immature neurons^29,30^. However, the mechanism that shifts the inhibitory/excitatory behavior in GABA neurons is not robust, a dysfunction in this neural mechanism may generate inhibitory neurons functioning as excitatory ones^31^. Besides, chronic stress can trigger this malfunction giving rise to epileptic seizures^32^.

On the other hand, two fundamental and dynamical properties of neural networks are 1/*f* fluctuations and synchronization. 1/*f* fluctuations (pink noise) represent the fractal temporal properties of neural networks that exhibit long-range memory. 1/*f* fluctuations have been found for mental tasks and spontaneous fluctuations recorded by electroencephalograph (EEG)^33,34^, magnetoencephalograph (MEG)^35^ and functional magnetic resonance imaging^36^. Notably, deviations from 1/*f* fluctuations have been found in some patients for diverse brain disorders and aging^37–45^. In addition, neural networks are very sensitive to 1/*f* fluctuations^46–48^.

Synchronization is the spontaneous organization that gives rise to collective neuronal firing^49–52^; synchronization is also the fingerprint of communication and processing in neural networks. Several authors report that healthy neural networks display critical synchronization, where a negative power-law distribution of global phase changes (or neural avalanches sizes) is observed, indicating the presence of few global phase changes and many local ones^53–58^. Other studies focused on brain activity have reported variations in the levels of synchronization in neuropathologies, either a reduction or an oversynchronization^59–64^. In the context of brain models, simulations show that inhibitory and excitatory hub neurons can drive the system from desynchronized to fully synchronized states^65–70^.

In 1950, Alan M. Turing wondered if brain dynamics operate in a critical stability regime^71^. Four decades later, some authors indicated the strong relation between 1/*f* fluctuations and a power law distribution of global neural changes^72,73^; then others showed the relation between simulated neural avalanches and 1/*f* fluctuations in brain^74,75^. Recently, using EEG and EMG, Palva *et al*. found that neural avalanches are strongly correlated with 1/*f* fluctuations^76^. Moreover, studies in simulated neural networks showed that 1/*f* signals are correlated with intermediate and critical synchronization, while Brownian or uncorrelated signals are related to either subcritical or supercritical states^77–80^. However, there is not a full understanding about the relation between dynamical and structural properties of neural networks, and the proportion of inhibitory and excitatory hub neurons in human brain networks. Here we study, by means of a hierarchical neural model, the interplay between inter-hub connectivity and the number of excitatory and inhibitory hubs in order to measure the system dynamics in terms of temporal autocorrelation and global synchronization.

## Methods

### Hierarchical network and rich-club organization

**Figure 1:**
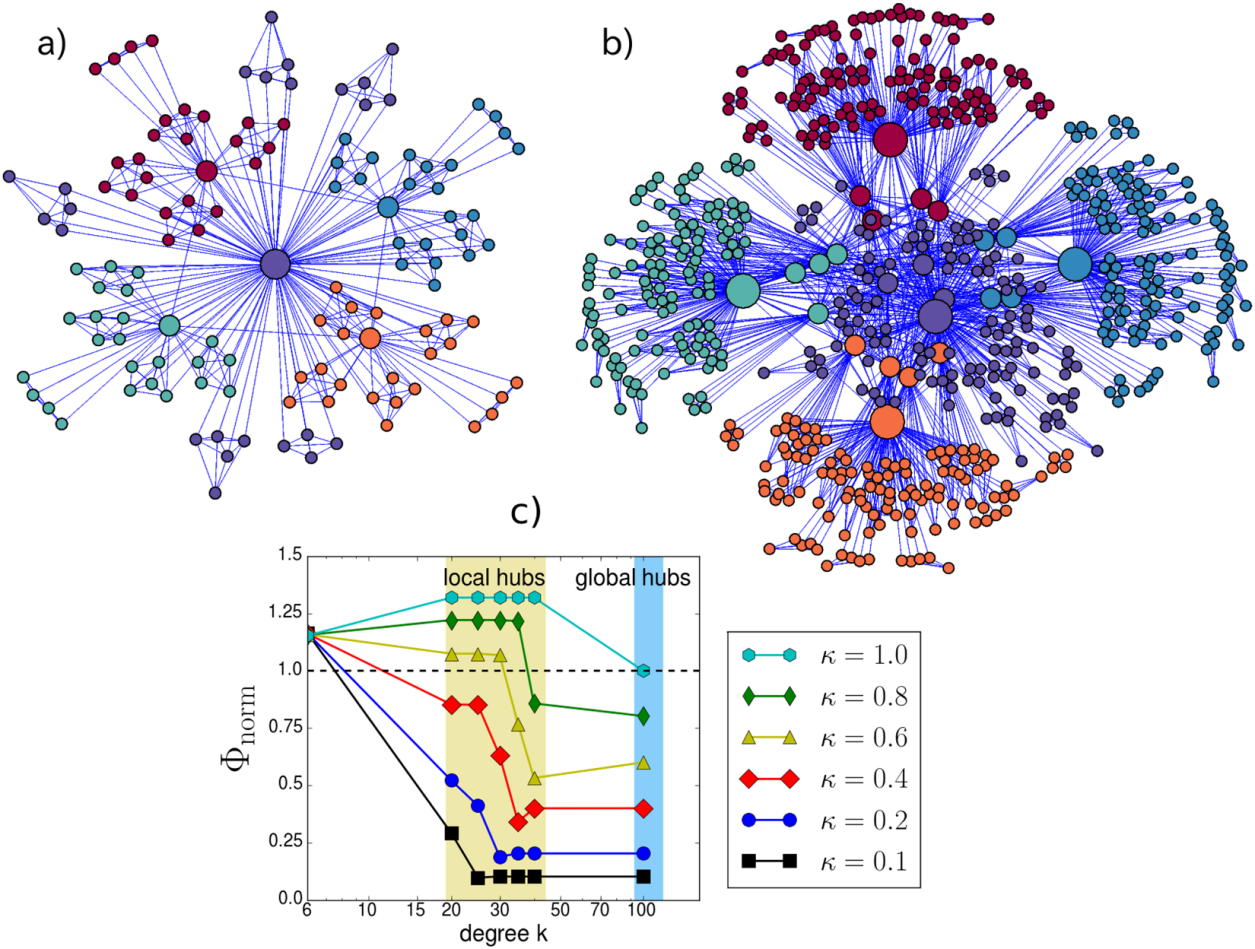
Representative plots of the hierarchical network model. (**a**) Network with 125 nodes at step 2 with a rich-club organization $\kappa =1.0$. Size of nodes indicates degree connectivity. The color of the nodes indicates the five different clusters comprising 25 nodes each. We defined two types of hubs: global hubs $k\ge 80$, and local hubs, which are connected to only one cluster (25 nodes) and to the rest of the hubs. (**b**) 5 replicas of networks at step 2, connected by hubs $\kappa =1.0$. The color of the nodes indicates the five different clusters comprising 125 nodes each. (**c**) Normalized rich-club coefficients ${{\rm{\Phi }}}_{norm}$ in terms of the $\kappa$ values.

The neurons are located in a hierarchical scale-free network proposed by Ravasz and Barabási^81^. The connectivity degree distribution *P*(*k*) of the model follows a negative power-law function $P(k)\sim {k}^{-\gamma }$, with $\gamma =2.1$. The first step consists in constructing a cluster of five linked nodes (a complete network); creating four replicas, and finally connecting four nodes of each replica cluster to one node in the first cluster (hub node); this results in a network of 25 nodes including a hub. The second step consists in replicating the first step four more times, and connecting the resulting 16 peripheral nodes to the hub node proposed in step one; the output consists in a network with 125 nodes and 5 hubs (Fig. 1a). The algorithm presented can be repeated indefinitely, where each step increases the number of nodes by a factor of 5. In this study, 5 network replicas, similar to the one created in step 2, were used to form a network with 625 nodes (see Fig. 1b). In our case, we consider that all edges are bidirectional. Specifically, we assumed that there are two weights for each bidirectional edge, one incoming and one outgoing link, and that each neuron sends an independent synaptic potential. All excitatory outgoing links activate and all inhibitory outgoing links send negative potential, which is in accordance with Dale’s law^82^. We also performed simulations establishing only unidirectional links to detect changes in the dynamics (see Supplementary Material). Next, each pair of hubs is connected with probability $\kappa$; such a deliberate setup allows hubs to communicate with each other. Two major types of hubs are defined: global hubs (with connections of $k\ge 100$ nodes) and local hubs (only connected to one group of 25 nodes and the rest of the hubs $20\le k\le 44$). A previous report has identified a lack of links between hubs in this network and its importance in modeling neural networks^83^. The rich-club organization is a main structural property of neural network systems where nodes with high connectivity degree tend to connect each other^7^. In order to detect the rich-club phenomenon in our configuration, we use the normalized rich-club coefficient ${{\rm{\Phi }}}_{norm}$^84^, which is defined as the number of edges between pairs of hubs normalized by the number of edges in a null model network. The null model comprises the same degree distribution with random connectivity between pairs of nodes. For ${{\rm{\Phi }}}_{norm} > 1$, the configurations show legitimate rich-club organization. Figure 1c shows rich-club coefficients of the model for different values of $\kappa$ and connectivity degree *k*.

### Izhikevich neuron model

We use the integrate-and-fire neuron model presented by Izhikevich^85^. The principal advantage of the model over others is that it reproduces many dynamic features of real neurons with low computational cost^86^. The model is a two-dimensional system of ordinary differential equations defined by the following equations:

1 $$\dot{v}=h(0.04{v}^{2}+5v+140-u+I+s)$$

2 $$\dot{u}=h(a(bv-u))$$

3 $${\rm{if}}\,v\ge 30\,{\rm{mV}},\,{\rm{then}}\{\begin{array}{l}v\leftarrow c\\ u\leftarrow u+d,\end{array}$$

where *h* is the size of the time step, *v* represents the membrane potential of the neuron, the notation $\dot{v}=dv/dt$, *u* represents a membrane recovery variable, which emulates the sodium – potassium pump. The parameters *a* and *b* represent the time scale and the sensitivity of the recovery variable *u*, respectively. The parameters *c* and *d*, represent the after-spike reset of *v* and *u* respectively. The value *I* represents a noisy thalamic input that is received for each of the neurons, and the value *s* represents the sum of the incoming potentials of its nearest neighbors when they fired. In our simulation, we consider a diverse repertoire of values for the parameters. For excitatory neurons: ${a}_{i}=0.02$, ${b}_{i}=0.2$, ${I}_{i}=5$, ${c}_{i}=-\,65+15{r}_{i}$ and ${d}_{i}=8-6{r}_{i}$, where *r*_*i*_ is a random variable uniformly distributed on the interval [0, 1], and *i* is the neuron index. If ${r}_{i}=0$, the neurons exhibit regular spiking, and if ${r}_{i}=1$, the neurons display chattering behavior. For inhibitory neurons: ${a}_{i}=0.02+0.08{r}_{i}$, ${b}_{i}=0.25-0.05{r}_{i}$, ${I}_{i}=2$, ${c}_{i}=-\,65$ and ${d}_{i}=2$. Here, if ${r}_{i}=0$, the inhibitory neurons show a slow recovery with low-threshold spiking; however if ${r}_{i}=1$, the neurons show fast spiking.

In the hierarchical model, the hubs represent 4% of all nodes; we variate the proportion of excitatory-inhibitory hub neurons to observe its dynamics, so we set the 5 most connected neurons or global hubs as either inhibitory (case 1) or excitatory (case 2). Next, we variate the proportion of excitatory-inhibitory local hubs and define the value $\eta$ as the probability of a local hub becoming inhibitory. For the rest of the nodes (96% of the total), we set 80% of them as excitatory and 20% as inhibitory.

In order to generate a time series of the evolution, we define the state of the system as,

4 $$S(t)=\frac{1}{N}\,\sum _{i=1}^{N}\,{v}_{i}(t),$$

where *N* is the total number of units. We consider time evolutions of the system comprising 625 neurons. After a transient period (8000 time steps) the state of the system is monitored for 10^4^ additional time steps.

### Time resolution and synaptic weight

It is well known that the best individual accuracy of the Izhikevich model is reached for time steps, *h*, less than 0.5 ms^87–89^. However, the original Izhikevich model^85^ uses the forward Euler integration with time steps of 0.5 and 1 ms, and other authors have used similar time steps for achieving collective and critical dynamics^77,80,90,91^ (see Table 1). A very recent study by Pauli *et al*.^92^, focused specifically on the reproducibility of the Izhikevich model, demonstrates that the model is highly sensitive to the integration time step. Namely, they observed dynamic changes in network dynamics while they were decreasing the time resolution from 1.0 ms to 0.1 ms. The authors also offer a guide to reproduce global dynamics at time steps of 0.1 ms, which is based on the increment of the synaptic weight *s* in relation to the corresponding average firing rate. Here we adopt Pauli *et al*.’s approach by looking for an average excitatory firing rate of 5 spikes per second (spks/s), which is an intermediate firing rate close to the ones used in the original Izhikevich model (2–7 spks/s)^93^. In addition, we use the 2nd-order Runge-Kutta (RK-2) method to implement the numerical integration of the differential equations with a time resolution of 0.1 ms (see Results Section for additional discussion about other time resolutions). Using the RK-2 with a time resolution of 0.1 ms can be considered as a high-quality simulation^89^. We also provide an online source of the model’s implementation, where the time evolution, a simulation in mp4 format, and the detrended fluctuation analysis can be obtained from a standalone script^94^.

**Table 1:** Parameters of spiking neuron models that reproduce global neural dynamics.

Authors	Model	time step	Indegree	Synaptic strength (mV)
Izhikevich^85^	2003	1 ms	1000	E = [0.0, 0.5], I = −[0.0, 1.0]
Izhikevich^93^	2006	1 ms	100	E = 6, I = −5
Lombardi *et al*.^80^	Integrate and fire	4 ms	3–100, scale-free	E = [0.15, 0.3], I = −[0.15, 0.3]
Massobrio *et al*.^90^	Izhikevich (2003)	1 ms	20–100, scale-free	E = 10, I = −6
Poil *et al*.^77^	Integrate and fire	1 ms	8–48	E = 0.011, I = −2
Wiles *et al*.^107^	Izhikevich (2003) with autapses	0.2 ms	100	E = 7, I = −6
Pauli *et al*.^92^	Izhikevich (2006)	0.1 ms	100	E = [50, 85], I = −[50, 85]

We listed the values of integration time step, average indegree and synaptic strength.

### Detrended Fluctuation Analysis

Detrended fluctuation analysis (DFA) is a reliable method to detect long-range time correlations in nonstationary time series^95^. DFA considers the following steps. First, the signal (in this case the state of the system, *S*(*t*)) is integrated; the resulting series (*y*_*i*_) is divided into windows of size *n* and for each window, a straight line is fitted to the points (*y*_*n*_). Next, the root-mean-square fluctuation is computed of the detrended sequence within each window: $F(n)=\sqrt{\frac{1}{{\mathscr{N}}}\,{\sum }_{i=1}^{{\mathscr{N}}}\,{[{y}_{i}-{y}_{n}]}^{2}}$. If a scaling function of the form $F(n)={n}^{\alpha }$ is present, then the correlation exponent *α* characterizes the original signal. It is known that $\alpha =0.5$ corresponds to white noise (uncorrelated signal), $\alpha =1.5$ corresponds to Brownian noise, and $\alpha =1$ corresponds to a long-range correlated process or 1/*f* noise. In this work, we obtain *F*(*n*) and *α* for the state of the system *S*(*t*) under different configurations.

### Global Lability of Synchronization

The aim of this work is to measure the avalanche activity among sets in the system in terms of excitatory/inhibitory hub proportion and rich-club connectivity. Here, we use the global lability of synchronization method^53^, which is suitable for the detection of local and global events of synchrony. An advantage of this measure over the standard avalanche analysis is that the definition of the avalanche size is based on a quiescent initial condition in the network, so some kinds of sustained firing or supercritical behaviors are difficult to measure using the avalanche analysis. Moreover, the lability of global synchronization allows us to detect a wide range of stability regimes. This measure is based on the rate of change in the number of phase-locked signals pairs between successive time steps. We generate 125 signals from the average local state (of membrane potentials *v*_*i*_) of clusters of 5 nodes. As in the original lability work, we assume that two signals are synchronized at certain time step if they satisfy two conditions: the absolute value of the phase difference, ${\rm{\Delta }}{\theta }_{i,j}(t)$, must be smaller than *π*/4; an index synchronization parameter, ${\gamma }_{i,j}(t)$, must indicate similar frequencies (${\gamma }_{i,j}(t) > \sqrt{1/2}$). The phase difference between two signals *s*_*i*_(*t*) and *s*_*j*_(*t*) is given by^96^

5 $${\rm{\Delta }}{\theta }_{i,j}(t)=arctan[\frac{{\tilde{s}}_{i}(t){s}_{j}(t)-{\tilde{s}}_{j}(t){s}_{i}(t)}{{s}_{i}(t){s}_{j}(t)-{\tilde{s}}_{i}(t){\tilde{s}}_{j}(t)}],$$

where ${\tilde{s}}_{i}(t)$ and ${\tilde{s}}_{j}(t)$ represent the Hilbert transform of *s*_*i*_(*t*) and *s*_*j*_(*t*), respectively. The Hilbert transform is defined as^96^:

6 $$\tilde{s}(t)=\frac{1}{\pi }P.\,V.{\int }_{-\infty }^{\infty }\,\frac{s(\tau )}{t-\tau }d\tau ,$$

where *P*.*V*. indicates the Cauchy principal value. The phase synchronization index is used to meet the second condition^96,97^:

7 $${\gamma }_{i,j}(t)=\sqrt{{[\frac{1}{v}\sum _{t}^{t+v}\cos {\rm{\Delta }}{\theta }_{i,j}(t)]}^{2}+{[\frac{1}{v}\sum _{t}^{t+v}\sin {\rm{\Delta }}{\theta }_{i,j}(t)]}^{2}},$$

where *v* is the size of the time window. We set $\nu =50$ time steps. For ${\gamma }_{i,j}=0$, there is a large frequency difference during the time window, while for ${\gamma }_{i,j}=1$, the phase difference is constant during the whole interval, indicating full synchronization. Next, we count the number of pairs of signals that are synchronized at time *t*:

8 $$M(t)=\sum _{i < j}\,[|{\rm{\Delta }}{\theta }_{i,j}(t)| < \pi /4\,and\,{\gamma }_{i,j}(t) > \sqrt{1/2}].$$

Finally, from the statistics represented by *M*(*t*), we calculate the square of the difference in the number of synchronized pairs between two successive time steps, representing the lability of global synchrony^53^:

9 $$\ell (t)=|M(t+1)-M(t){|}^{2}.$$

A large value of $\ell$ indicates a global synchronization/desynchronization process, whereas a small value indicates a local change in synchronization. Previous studies reported that systems operating at critical regimens, such as Ising and Kuramoto models, show negative power-law distributions of $\ell$ values^53,98^, and similar distributions were also found for human brain functional signals^53^.

## Results

### Integration time step, firing rate and synaptic weight

**Figure 2:**
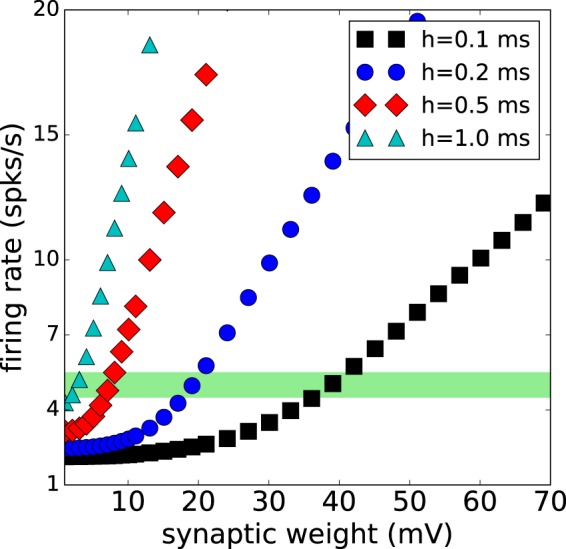
Firing rate vs. synaptic strength for different time resolutions. The plots correspond to the case 1, where all global hubs are inhibitory and all local hubs are excitatory ($\kappa =0.5$ and $\eta =0$). The shaded green bar indicates that by increasing the resolution (smaller *h*) to obtain a firing rate close to 5 spks/s, an approximate value of 40 for the synaptic weight is required.

To determine how robust our results are, first we address the question about the effects of changes of integration time steps on firing rate and on synaptic weight. Figure 2 shows the average firing rate of the network (Fig. 1b) vs. the synaptic weight for case 1 ($\kappa =0.5$ and $\eta =0$), for different time resolutions. The value of the parameter $\eta$ was selected to have a balanced inhibition/excitation hub activity, where all global hubs are inhibitory and all local hubs are excitatory. We observe that as the size of the time step increases, the model needs less synaptic weight in order to display the same firing rate. For instance, for time steps of 0.1 ms, a firing rate of 5 spks/s is reached approximately at 40 mV of synaptic weight. It is important to mention that the injected current is applied for a single time step. For this reason, the different curves are scaled versions of each other, with a scaling factor given by the ratio of the time resolutions. Thus, for *h* = 0.1 ms, the synaptic weight needs to be large to recover the dynamics of the original model.

Furthermore, other studies have found that synaptic strength and connectivity (degree) play a key role in driving the system toward criticality. As the synaptic strength increases, the system shows more synchronization^90^, and more connectivity is associated with more activity in the system^90^. Lastly, in order to achieve critical dynamics, synaptic weight and connectivity have to be large enough, otherwise, the system shows subcritical behavior^90^. In Table 1 we present the main characteristics of representative spiking neuron models, which are able to reproduce either global or critical dynamics.

For the first two models presented by Izhikevich, we notice that the decrease of incoming connectivity in the 2006 model was replaced by an increase in synaptic strength. A similar strategy is used in Massobrio’s study. In general, it is observed that for smaller time steps, the synaptic weight is bigger. A high time resolution reduces global activity, however, more incoming connectivity and more synaptic strength have been used to increase global activity.

### Time evolution and temporal autocorrelation

In order to characterize the signature of dynamical evolutions of specific configurations cases, where the hub activity plays an important role, first we investigate the time evolution of the hierarchical network model at step 1 governed by the Izhikevich model.

**Figure 3:**
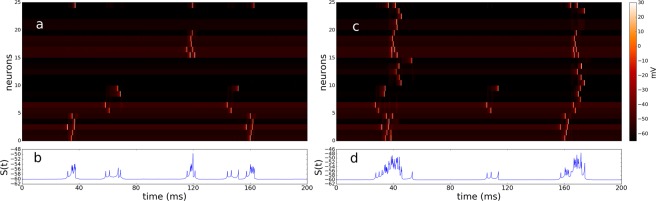
Time evolution and state of the system. (**a**) Time evolution of the system for the case where the local hub (neuron 25) is inhibitory. (**b**) The evolution of the state of the system *S*(*t*). (**c**) Time evolution for the case where the local hub (neuron 25) is excitatory. (**d**) The state of the system *S*(*t*).

In Fig. 3 we show representative time evolutions of the simplest configuration in our model. The network comprises 1 cluster with 25 units (one cluster in Fig. 1a). Each network posses 5 randomly selected inhibitory neurons, 19 excitatory neurons and 1 hub. For the case when the hub is inhibitory (Fig. 3a,b), we observe that the activation of neurons is transmitted to the hub, and the hub sends a negative feedback and rapidly stops the propagation of the firings. In contrast, for the case when the hub is excitatory (Fig. 3c,d), the activation of the excitatory hub promotes the activation of all units, giving rise to fully synchronized states.

**Figure 4:**
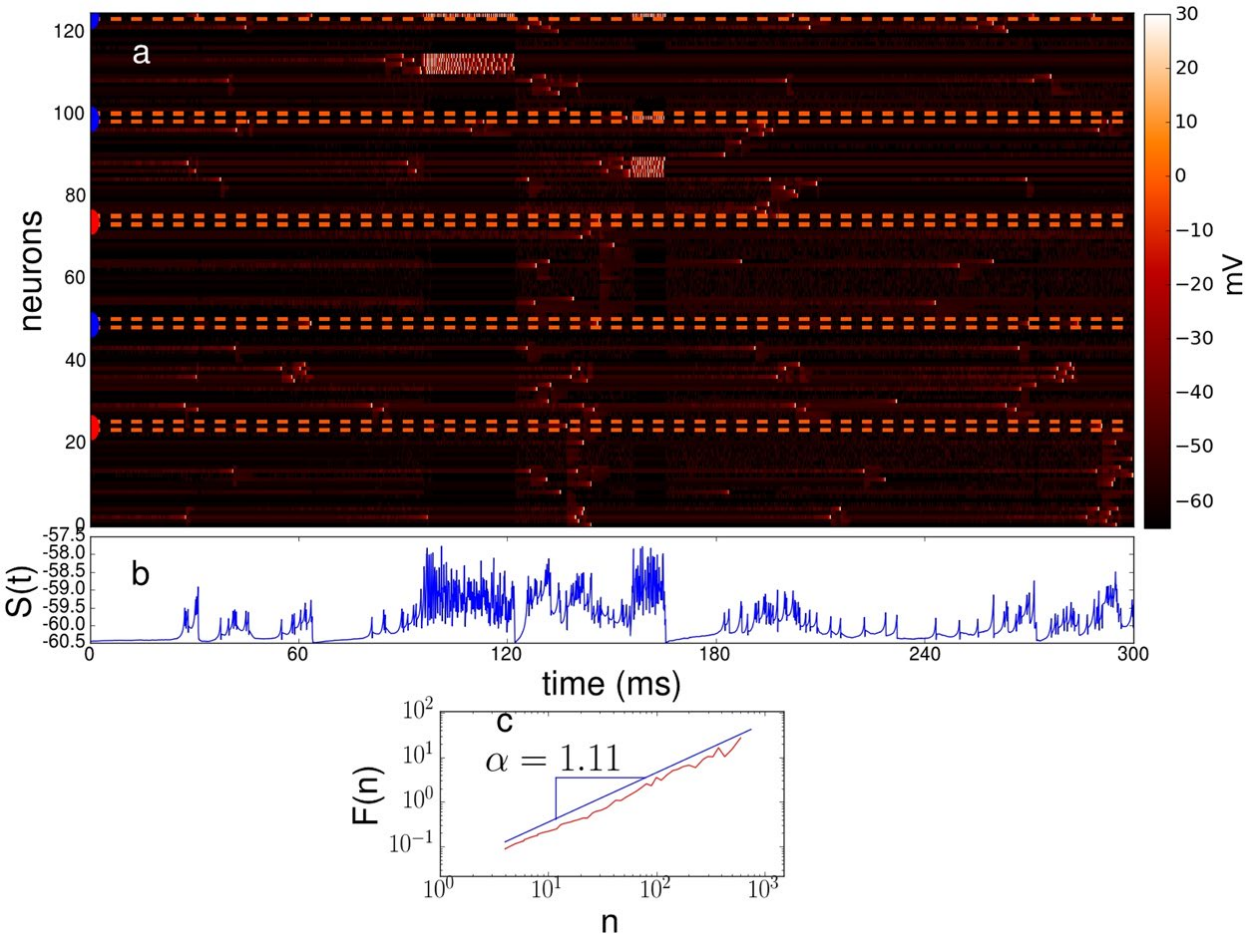
Time evolution and quantification of autocorrelations. Colored semicircles indicate either excitatory (red) or inhibitory (blue) behavior. (**a**) Time evolution of the system for the case where the most-connected node (#125) and two local hubs (node #50 and #100) are inhibitory, while node hubs #25 and #75 are excitatory; with a rich-club probability $\kappa =0.5$. The network size is $N=125$. Dash lines surround the temporal evolution of hubs. (**b**) State of the system for the evolution shown in (**a**). (**c**) Log-log plot of the fluctuation *F*(*n*) vs. the time scale *n*. Here the exponent *α* represents the type of correlations displayed by the system and it is related to the exponent *β* of the power spectrum of the fluctuations, $S(f)\propto 1/{f}^{\beta }$, by means of the relation $\beta =2\alpha -1$. The evolution of *S*(*t*) shown in (**b**) yields the scaling exponent $\alpha \approx 1.1$, indicating the presence of long-range correlations.

Next, we investigate the time evolution of the hierarchical network model at step 2 governed by the Izhikevich model. Figure 4a depicts the time evolution of the neural model where the most-connected node (node 125) and two local hubs (node 50 and 100) are inhibitory. In the figure, dash lines surround the temporal evolution of hubs, and also divides the 5 clusters of 25 neurons. For example, neuron 25 is a local excitatory hub which is connected with 20 neurons with the neuron indexes in the range 1–24.

We observe that the avalanche activity is present in terms of temporal activity of clusters, which are not stable and this fact contributes to the emergence of 1/*f* fluctuations in the *S*(*t*) dynamics (Fig. 4b,c). Previous findings indicate that there are several real and simulated systems operating at critical regimes which exhibit 1/*f* dynamics. The majority of these systems do not posses a long-tail connectivity distribution to show 1/*f* dynamics. However, the evidence of functional and structural connectivity suggest that the real topology of brain networks is characterized by the presence of hub regions, thus it is important to study the emergence of 1/*f* dynamics in the context of hub interconnectivity and functionality.

The activation of clusters (comprising 25 nodes) is mainly initiated by small clusters (comprising 5 nodes) with no hub nodes, then the activation is transmitted and amplified to other small clusters by excitatory hubs. This qualitative behavior is quite similar to the findings reported by Luccioli *et al*.^68^, where the excitatory hubs are reminiscent of connector and provincial hubs^8^. It is noteworthy that during the time evolution, the firing activity between the different clusters is correlated, suggesting that excitatory hubs play a central role in information transmission between clusters. Besides, we observe diverse activity patterns. In the literature, critical dynamics are related to high information transmission and the existence of many attractor states^8,55,99^. On the other hand, the global inhibitory hub controls the occurrence of large avalanches (Fig. 4a), suggesting the important role that inhibitory hubs (GABA neurons) play in giving rise to critical dynamics. To our knowledge, there is some evidence that integrate-and-fire neurons can display 1/*f* fluctuations^100–102^. Here Fig. 4 shows that a mixed population of inhibitory and excitatory hubs and an intermediate rich-club connectivity promote the deployment of 1/*f* fluctuations.

**Figure 5:**
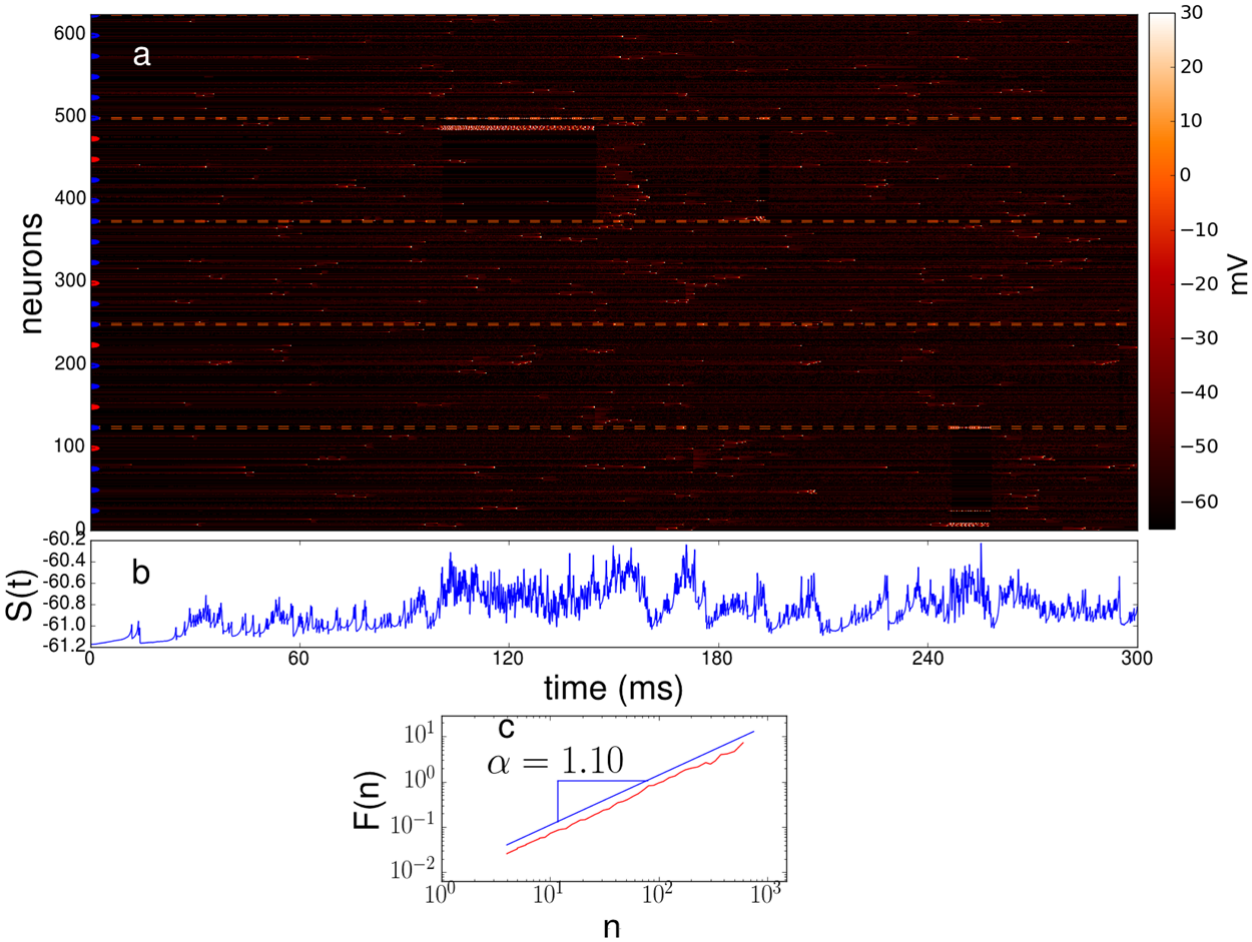
Time evolution and quantification of correlations for a network with 625 nodes. (**a**) Evolution of the case where the most connected nodes are inhibitory (case 1), with the fraction of local inhibitory hubs $\eta =0.75$ and a rich-club probability $\kappa =0.75$. (**b**) State of the system *S*(*t*) for the evolution in (**a**). (**c**) DFA analysis of *S*(*t*) yields a scaling exponent close to 1, indicating long-range correlations.

In order to test the robustness of the dynamics with respect to the number of neurons in the network, we repeat the temporal analysis with a set of 5 clusters with 125 units each. Now 5 replicas of the hierarchical network at step 2 with 625 nodes are considered, where 5 of them are global hubs and 20 are local hubs (see Fig. 1b). Two different cases are considered: global hubs are inhibitory (case 1) and global hubs are excitatory (case 2). The time evolution of case 1, where global hubs are inhibitory ($\kappa =0.75$ and the fraction of local inhibitory hubs $\eta =0.75$) is shown in Fig. 5a. The time evolution shows that the activation of big clusters (comprising 125 nodes) is correlated with other big clusters within and outside the 5 replicas (see Supplementary Material for the behavior of the correlation exponents in terms of the number of clusters). Interestingly, the state of the system exhibits 1/*f* fluctuations (Fig. 5b,c). We observe that the dynamics corresponding to a mixed population of inhibitory and excitatory local hubs with inhibitory global hubs is reminiscent of the case 1 with 1 cluster.

**Figure 6:**
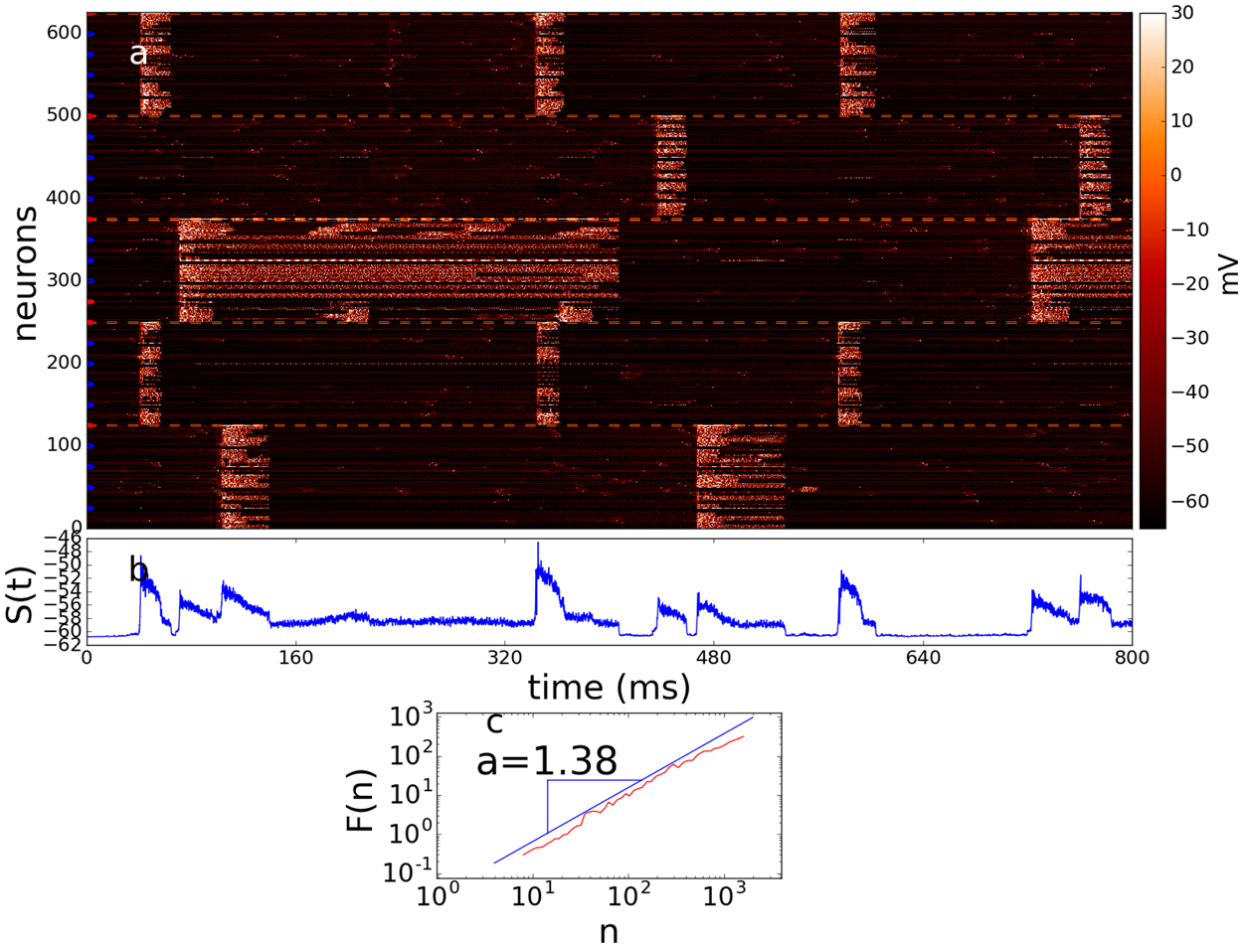
Time evolution and quantification of autocorrelations. (**a**) Representation of time evolution for case 2, $\kappa =0.15$ and $\eta =0.9$. (**b**) State of the system *S*(*t*) for the evolution shown in (**a**). (**c**) Log-log plot of the fluctuation *F*(*n*) vs. the time scale *n*. Data shown in (**b**) yields the exponent value $\alpha \approx 1.38$.

The time evolution of case 2, where global hubs are excitatory ($\kappa =0.15$ and $\eta =0.9$) is shown in Fig. 6a. We observe a global correlated behavior, where the vast majority of neurons in the big clusters are synchronized. We also observe that 1/*f* fluctuations get destroyed and the state of the system *S*(*t*) displays Brownian-like noise with $\alpha \approx 1.38$ (Fig. 6b,c).

**Figure 7:**
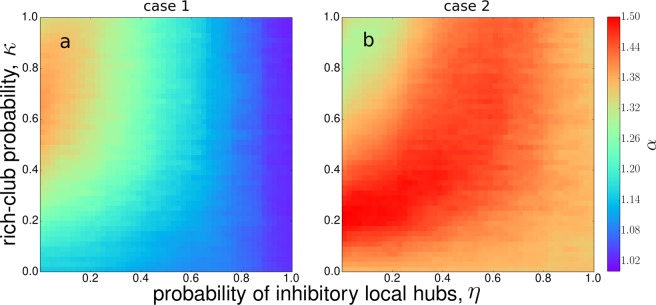
Phase space $\kappa$ vs. $\eta$ for scaling temporal exponents *α*. (**a**) Case 1: global hubs are inhibitory. (**b**) Case 2: global hubs are excitatory. Each pixel value is an average over 50 independent runs.

To strengthen our results, we systematically calculate the scaling temporal exponent *α* in terms of the rich-club connectivity $\kappa$ and the excitatory/inhibitory hub proportion $\eta$. The phase space of the scaling temporal exponent *α* is shown in Fig. 7. For the hierarchical network in case 1 (Fig. 7a), the phase space mainly exhibits 1/*f* noise ($\alpha \approx 1.0$). However, for $\eta  < 0.3$ and $\kappa  > 0.4$, we observe a region where the scaling exponent is $\alpha  > 1.3$, indicating Brownian-like fluctuations. When the number of excitatory hubs is dominant (low value of $\eta$) and the rich-club connectivity increases, the system becomes more activated, which is reflected in the increment of the *α* exponent. For intermediate values of both $\kappa$ and $\eta$, global inhibitory hubs control the production of global avalanches and this fact is reflected in the deployment of 1/*f* noise, also in agreement with the results described in Fig. 4. In contrast, for case 2 (Fig. 7b), the phase space mostly exhibits exponent values close to Brownian noise ($\alpha \approx 1.5$). Notably, when the hierarchical network structure is destroyed but the same incoming degree distribution is preserved, the model still displays a variety of dynamical behaviors, but with a more widespread presence of Brownian-like noise, indicating a more activated behavior with short-range correlations (see Supplementary Material). In addition, considering only unidirectional links promotes the decrement of *α* exponents values and more presence of 1/*f* noise (see Supplementary Material). The fact that the phase space gets slightly altered by destroying the clustering structure or considering unidirectional links reinforces the importance of the rich-club connectivity and the inhibitory/excitatory ratio.

### Global lability of synchrony

**Figure 8:**
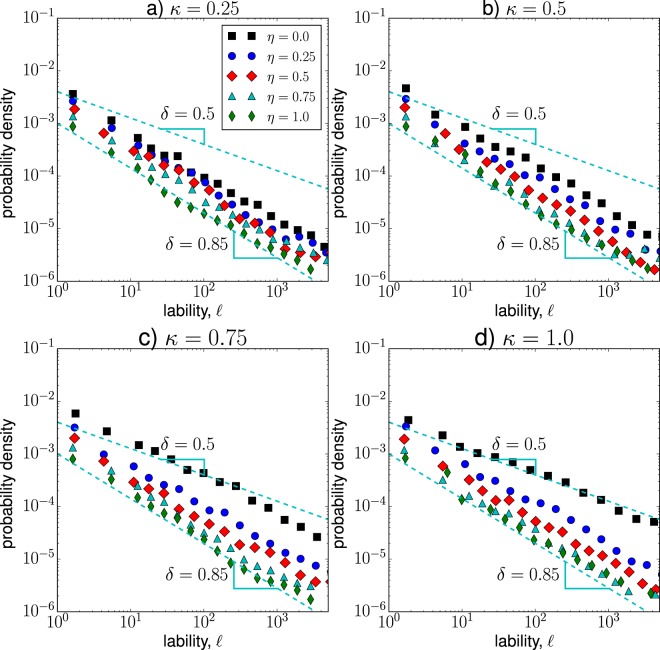
Probability density function of lability values for case 1 and different values of $\kappa$ and $\eta$. Each function represents the result of 100 independent runs.

Figure 8 shows the probability density of lability values for case 1 and different values of $\eta$ and $\kappa$. We observe that the probabilities follow a power-law $G(\ell )\sim {\ell }^{-\delta }$, with exponent values within the range $0.5\le \delta \le 0.9$. For the estimation of the power-law exponent, we used the method proposed by Hanel *et al*.^103^, which is suitable for the estimation of power-law exponents that are located within the range $0 < \delta \le 1.0$. For example, when $\kappa =1.0$ and $\eta =0.25$, the synchronization exponent is $\delta =0.88\pm 0.04$, where ±0.04 indicates the mean square error of the power-law fit^103^; while for $\kappa =1.0$ and $\eta =0.25$, the corresponding exponent is $\delta =0.83\pm 0.08$. Interestingly, for these intermediate values of $\kappa$ and $\eta$ we also observe long-range correlated temporal dynamics in Fig. 7a. Moreover, as the fraction of inhibitory neurons increases (a larger $\eta$), the frequency of lability events decreases, indicating a reduction in the occurrence of local and global synchronization changes. For $\eta =0$ (mostly excitatory hubs) and high values of $\kappa$, the *δ* exponents lie within the range from 0.66 to 0.58, indicating a legitimate power-law behavior. These values of $\kappa$ and $\eta$ correspond to Brownian fluctuations in Fig. 7a. Although there is not a specific range for *δ*-values directly associated to critical states, there have been reported critical synchronization exponents within the range $0.5\le \delta \le 1.0$^53,78,98^

**Figure 9:**
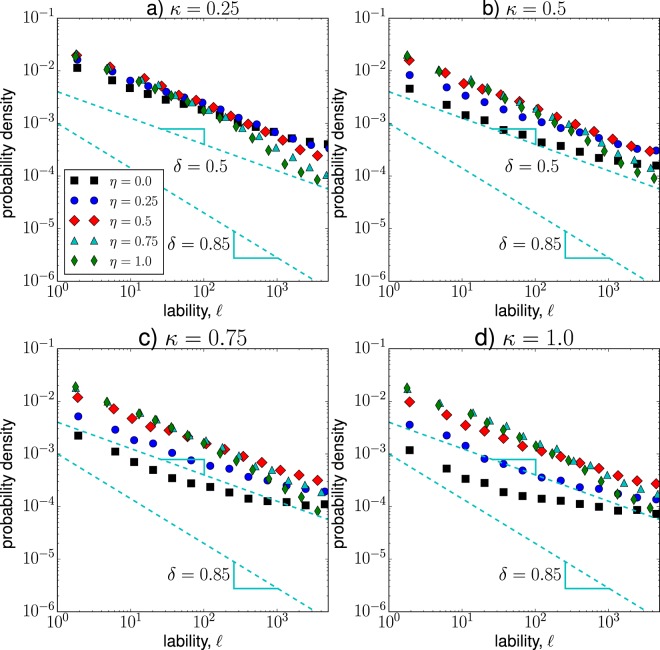
Probability density function of lability values for case 2 and different values of $\kappa$ and $\eta$. Each function represents the result of 100 independent runs.

In Fig. 9, we show the probability density of lability values for case 2. We observe that for low hub interconnectivity, $\kappa =0.25$, the majority of distributions follow a power-law function with exponent $\delta \approx 0.5$. These distributions are similar to the ones found in case 1 ($\eta =0.0$ and $\kappa \ge 0.75$), suggesting that for these configurations the synchronization exponent $\delta \approx 0.5$ is associated with Brownian fluctuations in the state of the system (see Fig. 7a). Moreover, when the majority of hubs are excitatory and the rich-club connectivity is high ($\kappa =1.0$ and $\eta =0.0$), the lability distribution displays an almost flat behavior with exponent $\delta \approx 0.24\pm 0.06$, where local and global phase changes take place with similar probabilities.

## Discussion

Our current understanding of brain function is directly linked to network topology and dynamics occurring within the brain, which have been explored in detail by network neuroscience. However, limitations imposed by recording tools - which provide low quality readings of the brain - continue to generate gaps of our understanding of how brain networks operate. Our simple neural model with scale-free topology is able to display a variety of dynamical patterns, levels of synchronization and temporal autocorrelation in terms of a hub’s inter-connectivity and excitatory/inhibitory proportions. Our results show that 1/*f* fluctuations are closely related to a power-law distribution of global phase changes, which emerge when there is a mixed population of inhibitory and excitatory hubs for broad interval values of rich-club connectivity. However, when the majority of hubs are excitatory and the rich-club connectivity is high enough, the system displays Brownian fluctuations, indicating a high frequency of global phase changes. These results are consistent with previous findings about the fact that identified hub neurons in Human and C. elegans neural networks are principally inhibitory (GABA interneurons)^24,104^. It is worth mentioning that similar critical behavior displaying 1/*f* noise, critical avalanche distribution and critical synchronization have been also detected in regular and small-world configurations^78,105,106^, and that in many cases the existence of 1/*f* noise depends on the balance between excitatory and inhibitory interaction^77,80^. However, much evidence suggests that real neural networks posses units that exert more influence than others, and that this influence is reflected in the connectivity of the units and synaptic strength. Our study based on the high resolution Izhikevich model comprises large synaptic weights that are higher than those reported in real electrophysiological recordings. Further works should incorporate the problem of the duration of synaptic release in order to reduce the weights and generate a model with more realistic parameter values.

Furthermore, our results are concordant with previous findings about the fact that a scale-free distribution of degree connectivity promotes critical distribution of avalanches; configurations with only excitatory hubs are unable to show criticality^90^ and 1/*f* noise appears when a mixed population of inhibitory and excitatory hubs are present^77^. Moreover, the disruption of rich-club connectivity promotes the desynchronization of the system and the reduction of the temporal autocorrelation exponent, while the total shift from inhibitory to excitatory behavior of hubs destroys 1/*f* fluctuations and power-law synchronization. Our results also show that the disruption of the hierarchical connectivity promotes the increase of global activity and the presence of Brownian fluctuations, on the other hand, the unidirectional link configuration facilitates the decrement of *α* exponents and the existence of 1/*f* noise.

Although different mechanisms and models have been proposed to generate 1/*f* noise^72–75^, it is important to remark that few models are able to generate long-range correlations with critical power-law synchronization based on large number of communicating units or neurons^77,78^. In these models, the topology and a balanced excitation/inhibition population of units seem to play a important role for an efficient functional communication between different parts of the network^80,90^. In the context of neurophysiological systems, mental illnesses like epilepsy, schizophrenia and Alzheimer have been associated to alterations in the rich-club connectivity or the failure of hub responses. In addition, we demonstrate that, in the context of our model, ingredients like rich-club connectivity, inhibitory global hubs and excitatory local hubs are relevant to generate a vast repertoire of spatio-temporal patterns, including 1/*f* fluctuations and critical synchronization. In the context of brain studies, which focus on network topology and dynamics, our results indicate that the rich-club connectivity and the hub inhibitory/excitatory population are important properties, which may help to understand the variety of temporal correlations and synchronization levels reported in brain dynamics.

## Supplementary information

Supplementary Material
