# Supplementary material for: Critical synchronization and 1/f noise in inhibitory/excitatory rich-club neural networks
Source: Sci Rep. 2019 Feb 4;9:1258. doi: 10.1038/s41598-018-37920-w (PMC6361933; doi:10.1038/s41598-018-37920-w)
Supplement: Supplementary file 1 — Supplementary Material [file 41598_2018_37920_MOESM1_ESM.pdf]

Supplementary material

"Critical synchronization and 1/ f noise in inhibitory/excitatory rich-club neural networks"

D. Aguilar-Velázquez and L. Guzmán-Vargas
Unidad Profesional Interdisciplinaria en Ingeniería y Tecnologías Avanzadas
Instituto Politécnico Nacional, Ciudad de México, México

Numerical integration

The Izhikevich neuron model consists in a two-dimensional system of ordinary differential equations defined as:

$$\dot{v} = h(0.04v^2 + 5v + 140 - u + I + s) \tag{1}$$

$$\dot{u} = h(a(bv - u)) \tag{2}$$

$$\text{if } v \geq 30\text{mV}, \text{ then} \begin{cases} v \leftarrow c \\ u \leftarrow u + d. \end{cases} \tag{3}$$

We used the second-order Runge-Kutta method to implement the numerical integration of Eq. (1) and Eq. (2)

$$A_1 = h\,(0.04v^2 + 5v + 140 - u + I + s) \tag{4}$$

$$B_1 = h\,a(bv - u) \tag{5}$$

$$A_2 = h\,[0.04(v + \frac{A_1}{2})^2 + 5(v + \frac{A_1}{2}) + 140 - (u + \frac{B_1}{2}) + I + s] \tag{6}$$

$$B_2 = h\,a[b(v + \frac{A_1}{2}) - (u + \frac{B_1}{2})] \tag{7}$$

$$\dot{v} = A_2 \tag{8}$$

$$\dot{u} = B_2, \tag{9}$$

where A_i and B_i, i=1,2, represent the increments in the Eurler's method. See main text and online information for details of the other parameters (Implementation of the Izhikevich neural-network model, https://github.com/danielvelaguil/Hierarchical-Izhikevich-model.)

Disrupted hierarchical organization

We also performed simulations by randomly changing the direction of the incoming links, destroying the clustering and the hierarchical organization, but keeping the same incoming degree distribution (see Figure 1). For this configuration, the information can be transmitted to the entire system not only by the hubs but also by any node regardless of its connectivity. It is expected that this rapid communication between different parts of the network would produce a more activated behavior. The phase space defined by (η,κ) in case 1 (Figure 1 left) shows a variety of dynamical behaviors, but notably the region corresponding to Brownian noise is larger compared to that observed in the original case with clusters and hierarchies. It is also observed that exponents $\alpha < 1.1$ are almost not present in the phase space. This feature is likely due to the fact that now the information transmission occurs among units located in different parts of the network and not only among hubs, like in the hierarchical configuration. The case 2 (Figure 1 right) mainly shows Brownian fluctuations.

Temporal α exponents in networks with unidirectional links

We obtained the temporal autocorrelation exponents, α, for the hierarchical network establishing only unidirectional links (see Figure 2). The results show that the dynamics of the system, represented by $S(t)$, exhibits a significantly decrease in the α exponent values. For case 1 (Figure 2 left), we observed that the region that displays $1.0 < \alpha < 1.2$ is larger compared with the case of bidirectional configurations (Figure 7 of the main text); Brownian fluctuations are almost not present.

For case 2 (Figure 2 right), the region with Brownian fluctuations shows a reduction in comparison with bidirectional case. The vast majority of the phase space shows $1.2 < \alpha < 1.3$. In general, the effect of changing the bidirectional links to unidirectional ones is to reduce the correlation exponent values (less activated behavior), which is likely related to the fact that, in the case of unidirectional links, the propagation of information is relatively slow compared to the bidirectional case.

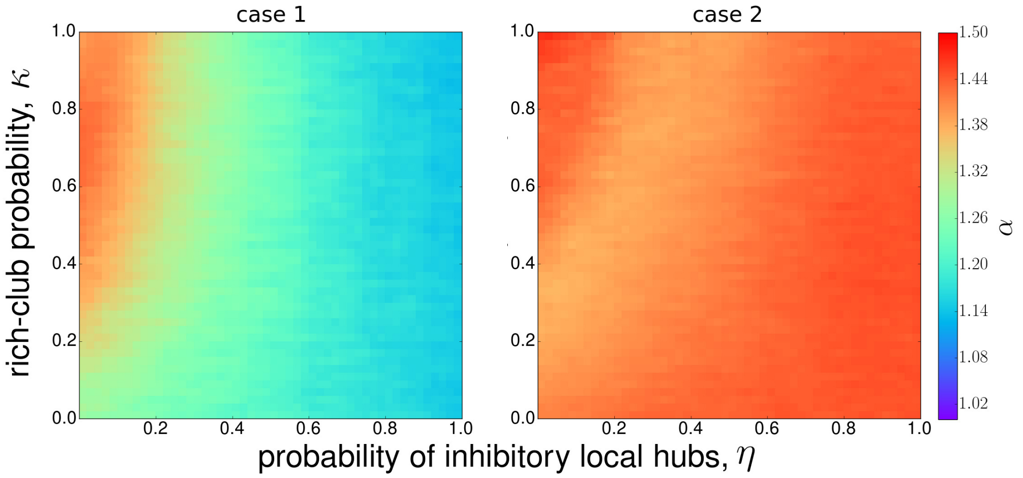

Figure 1. Phase space of η vs. κ for configurations with random direction of links. Temporal α exponents for case 1 (left). Case 2 (right).

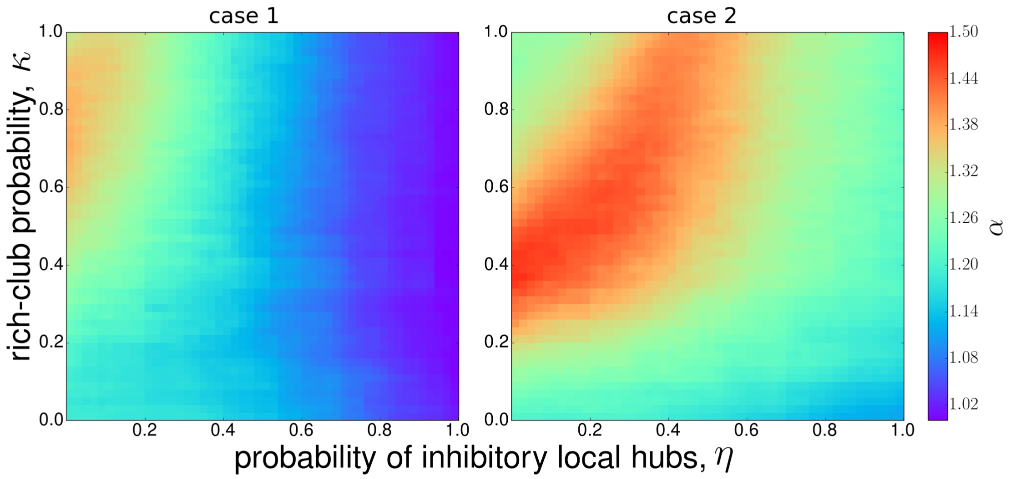

Figure 2. Phase space of temporal α exponents for configurations with unidirectional links.

Network size and correlation exponents

We show the behavior of the α-correlation exponent for two representative cases, which display $1/f$ and Brownian dynamics, respectively. We observe that the correlation exponent does not suffer significant changes (see Figure 3).

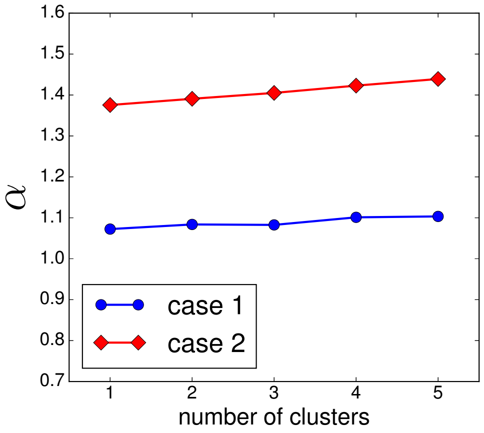

Figure 3. Temporal α exponents for two configurations: case 1 ($\kappa = 0.5$ and $\eta = 0.75$) and case 2 ($\kappa = 0.5$ and $\eta = 0.75$).
